# Pressure Reveals Unique Conformational Features in Prion Protein Fibril Diversity

**DOI:** 10.1038/s41598-019-39261-8

**Published:** 2019-02-26

**Authors:** Joan Torrent, Davy Martin, Sylvie Noinville, Yi Yin, Marie Doumic, Mohammed Moudjou, Vincent Béringue, Human Rezaei

**Affiliations:** 1 Institut National de la Recherche Agronomique, UR892, Virologie Immunologie Moléculaires, F-78350 Jouy-en-Josas, France; 2 Sorbonne Universités, UPMC Univ Paris 06, CNRS, UMR8233, MONARIS, Université Pierre et Marie Curie, F-75005 Paris, France; 3 Sorbonne Universités, Inria, UPMC Univ Paris 06, CNRS, UMR7598, Lab. J.L. Lions, F-75005 Paris, France

## Abstract

The prion protein (PrP) misfolds and assembles into a wide spectrum of self-propagating quaternary structures, designated PrP^Sc^. These various PrP superstructures can be functionally different, conferring clinically distinctive symptomatology, neuropathology and infectious character to the associated prion diseases. However, a satisfying molecular basis of PrP structural diversity is lacking in the literature. To provide mechanistic insights into the etiology of PrP polymorphism, we have engineered a set of 6 variants of the human protein and obtained PrP amyloid fibrils. We show that pressure induces dissociation of the fibrils, albeit with different kinetics. In addition, by focusing on the generic properties of amyloid fibrils, such as the thioflavin T binding capacities and the PK-resistance, we reveal an unprecedented structure-barostability phenomenological relationship. We propose that the structural diversity of PrP fibrils encompass a multiplicity of packing defects (water-excluded cavities) in their hydrophobic cores, and that the resultant sensitivity to pressure should be considered as a general molecular criterion to accurately define fibril morphotypes. We anticipate that our insights into sequence-dependent fibrillation and conformational stability will shed light on the highly-nuanced prion strain phenomenon and open the opportunity to explain different PrP conformations in terms of volumetric physics.

## Introduction

Prions are *in vivo* pathogens consisting of homomeric complexes of misfolded host cellular prion protein (PrP^C^) that self-propagate by a process of seeded polymerization^1,2^. These proteopathic seeds, commonly termed PrP^Sc^, are characterized most often as amyloid-like fibrils^3,4^.

Amyloid assemblies have a common cross β-structure with strands oriented perpendicularly to the fibril axis. However, these polymers can exhibit a plethora of structurally distinct conformations, suggesting a high level of polymorphism^5,6^. For instance, it is increasingly thought that the various and highly specific quaternary structural features of PrP fibrils underlie the different biochemical properties (i.e. fracture toughness, size, chemical and/or protease stability) of prions isolated from infected tissues as well as the typical pathogenic and phenotypic traits they elicit in the host. This gives rise to what is known as prion strains^7,8^. However, gathering structural information on the presumed myriad of PrP quaternary structure topologies remains a challenge.

Since adequate purification and structural characterization of highly-infectious preparations of prions from mammalian brain has proved difficult^9,10^, a more promising approach to study the conformational basis of PrP superstructural variation may come from progress in the understanding of fibrillar assemblies generated *in vitro*^11^. The ability to make *in vitro* fibril amyloids, at will, from purified recombinant PrP (recPrP) provides a model system where the structural/functional properties of these PrP assemblies can be elucidated. Knowing how PrP fibril diversity is related to the quaternary structure could aid in understanding how multiple prion strains are generated and stabilized from a unique native fold, and their physical relationship with infectivity.

Here we sought to obtain conformationally different human recPrP fibrils in order to decipher their underlying physical and structural properties. We provide conclusive evidence that a wide superstructural range of human PrP fibrils can be obtained by single amino acid substitutions designed to mimic several *PRNP* gene polymorphisms. Even if the various PrP proteins display structural homogeneity in the monomeric state, their assembly resulted into an array of distinct fibril types with distinguishable physicochemical properties. By studying the pressure-induced dissociation kinetics of the resulting PrP fibrils, we define a novel key property characterizing their assembly landscape: the fibril barostability. We report for the first time a direct correlation existing between the fibril pressure response and some of its generic properties, such as the thioflavin T (ThT) binding capacities and the PK-resistance. In addition, by assuming that the system volume changes accompanying PrP fibril dissociation are at the basis of pressure effects, the study raises the possibility that the fibril type-dependent protein volume defines the prion strain phenomenon. Finally, by focusing on the most contrasting PrP fibrils and using different seeding conditions, we report that the *in vitro* propagation efficiency is strongly influenced by the highly specific quaternary structural features.

## Results

### PrP amino acid substitutions distinctly affect amyloid fibril superstructure

**Figure 1:**
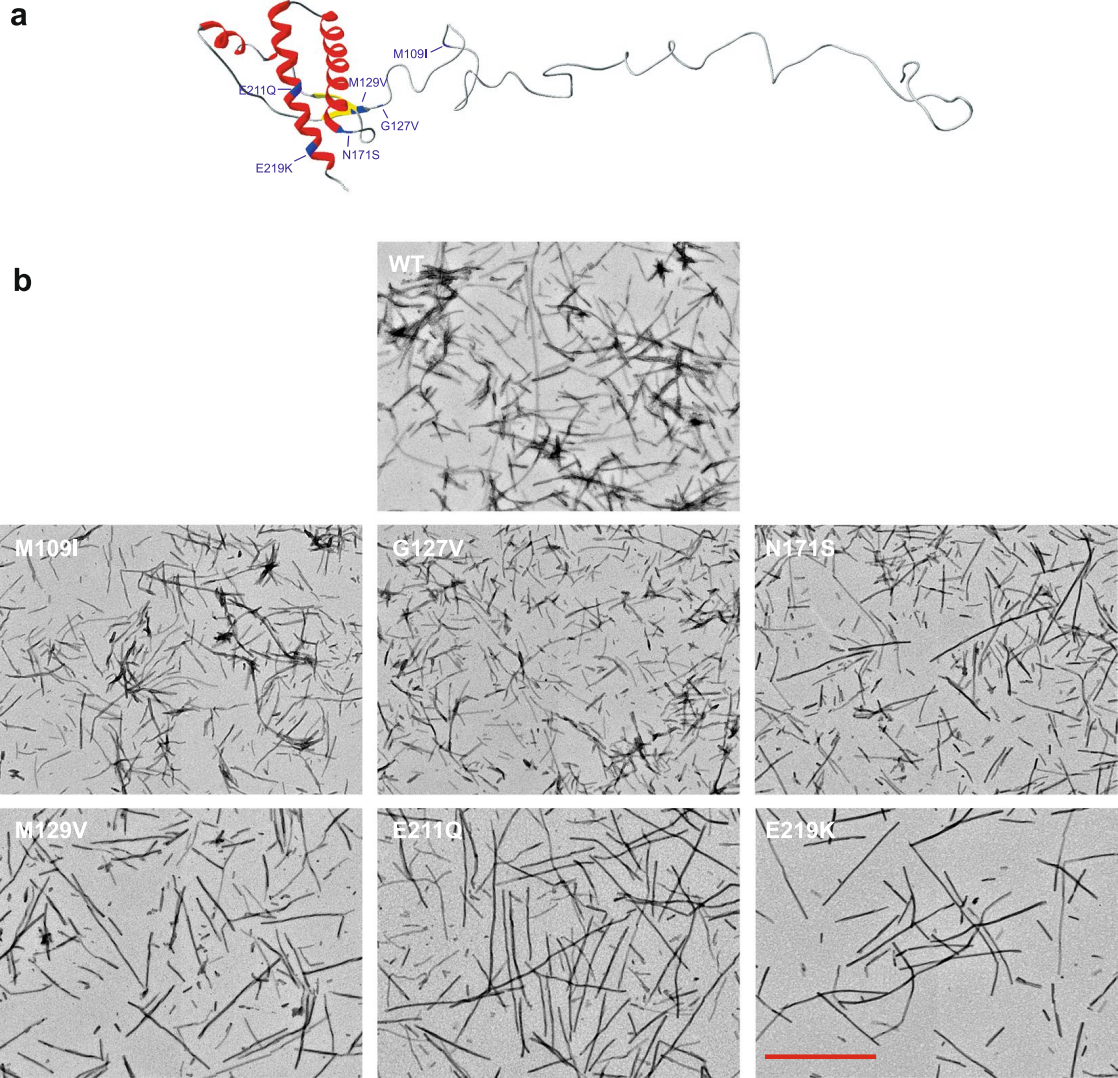
The human PrP forms and macrostructure of the PrP Fibrils. (**a**) Schematic representation of the three-dimensional structure of PrP showing the location of the substituted residues. The image was produced using Swiss-Pdb Viewer software and the crystallographic structure 1TQB with a reconstituted N-terminal disordered segment. (**b**) Negative-stained transmission electron micrographs of WT and variant PrP fibril forms as indicated. Scale bar, 1 μm.

We purified the wild-type full-length human PrP and a series of 6 variant forms that differ in single-point amino acid substitutions. These amino acid replacements correspond to the natural *PRNP* gene polymorphisms in humans G127V, M129V, N171S, E211Q, E219K, and in bank vole M109I (Fig. 1a). Circular dichroism (CD) spectroscopy revealed invariant secondary structure among the soluble native PrP variants (Supplementary Fig. 1).

Amyloid fibrils were then formed according to the method developed by the group of Baskakov and co-workers^11^. Analysis by transmission electron microscopy (TEM) confirmed their presence in all PrP samples (Fig. 1b). Preliminary visual inspection revealed variations in the characteristic fibrillar appearance (i.e. fibril length, fibril clumping) among different PrP variants, independently to the inherent fibril polymorphism observed either in a single fibril preparation or between sample replicates. Thus, we demonstrate that fibrillar diversity can be triggered by amino acid substitution.

**Figure 2:**
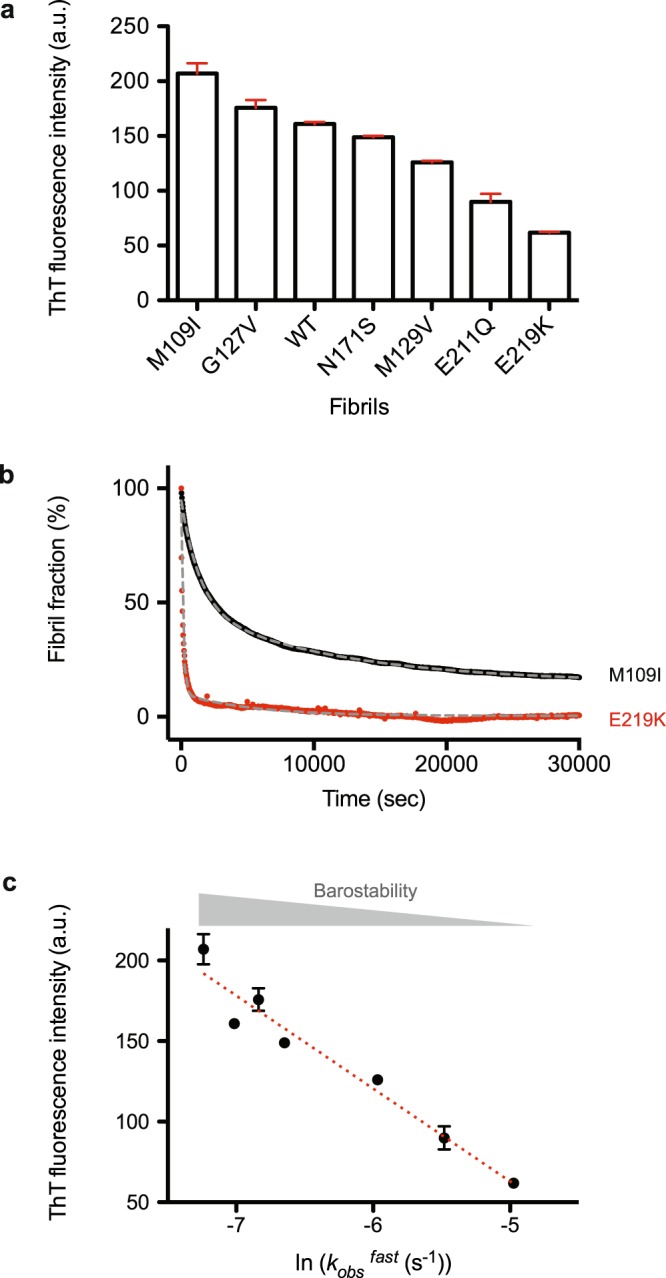
ThT-binding capacities of the PrP fibrils correlate with their barostability. (**a**) Binding of ThT to WT and variant PrP fibrils forms. ThT fluorescence emission maxima of each set of data is the mean value ± S.E of quadruplicate measurements. (**b**) Pressure-induced PrP fibril dissociation after a rapid increase of pressure to 300 MPa. The structural change kinetics for M109I (black line) and E219K (red line) were recorded as a decrease in light scattering. Fibril fraction was calculated from the amount of resolubilized PrP rescued after the pressure treatment, as judged from the absorbance at 280 nm of the supernatants obtained after removing fibrils by centrifugation. Dashed lines, linear fits to the data. a.u., arbitrary units. (**c**) Changes in the observed ThT-binding capacities of the PrP fibrils as a function of PrP fibril barostability. The dotted line shows the best fit to a linear equation (r = 0.937). Note: some error bars are on the order of the graph point size.

Analysis of the ThT fluorescence emission intensity of the various fibril samples identified a distinct spectral response. According to the ThT fluorescence associated with fibrillar amyloids, the different variant PrP fibril forms can be ordered as follows: M109I > G127V > WT > N171S > M129V > E211Q > E219K (Fig. 2a). This inter-sample variability strongly suggest that fibril multiple distinct appearances induced by single amino acid substitution affects the binding of such amyloid-specific dye. Propagation of M109I and E219K fibrils (the most divergent fibrils) by one round of homologous seeding resulted in the formation of “daughter” fibrils with similar ThT binding characteristics as the “mother” fibrils. Accordingly, even though an intergenerational evolution was observed, the difference in fluorescence intensity between both mutated fibrils was accurately maintained (Supplementary Fig. 2). These results led us to conclude that human PrP fibrils formed from at least two mutational variants retain their ThT-binding capacities with high fidelity. Furthermore, they dismiss the possibility that the changes observed are the results of the inherent heterogeneous nature of the amyloidogenesis process, and supports the hypothesis that they arise in response to amino acid replacement.

### The amyloid fibril pressure response correlates with the thioflavin T binding and PK-resistance properties

To find additional evidence for a structural polymorphism between each PrP fibril sample, we characterized the fibril dynamics by following their response to externally imposed pressure perturbations. To obtain a quantitative parameterization of the fibril barostability, the kinetics of the quaternary structure transitions were measured by monitoring light scattering after a fast increase (within 30 s) of pressure to 360 MPa at 25 °C (Fig. 2b) (Supplementary Fig. 3). Two exponential-decay kinetics were obtained. From the faster component of the decay, the relaxation times (τ_fast_ = *1/k*_*obs*_) varied between approximatively 2 and 23 min, depending on the fibril type, with M109I being the most resistant and E219K the most sensitive to pressure dissociation. Among the PrP fibrils, we found a linear correlation (r^2^ = 0.92) between *k*_obs_^fast^ values and the specific ThT fluorescent yield (Fig. 2c). In addition, the amount of resolubilized PrP rescued from the fibrillar form after the pressure treatment varied depending on the fibril type, as judged from the absorbance at 280 nm of the supernatants obtained after removing insoluble aggregates by ultracentrifugation. Thus, we observed a similar relationship for the ThT fluorescence intensities and the extent of dissociation of each fibril type, albeit with a lower degree of correlation (r^2^ = 0.71) (Supplementary Fig. 4). The kinetic profiles were found to be highly reproducible, even upon assaying two independent PrP fibril preparations obtained from a different stock of PrP WT sample (Supplementary Fig. 5), indicating that the different pressure behaviour observed among PrP fibrils is readily controlled by single amino acid substitution.

**Figure 3:**
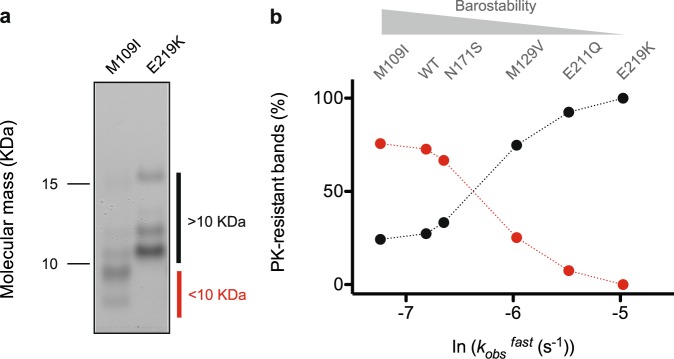
PrP PK-resistant core varies among PrP fibrils in accordance with their barostability. (**a**) Cropped silver staining gel showing the digestion profile of the PK-resistant core of M109I and E219K PrP fibrils. Full-length gel is shown in Supplementary Fig. 6. (**b**) Changes in the observed PrP PK-resistant bands as a function of PrP fibril barostability. The percentage of low molecular weight bands (<10 KDa, red) *vs* high molecular weight bands (>10 KDa, black) was calculated from a densitometric analysis. As shown, each pair of data points on the scatter plot represents a distinct PrP fibril.

Another characteristic of polymorphic PrP fibrils is that PK generates a different pattern of polypeptide fragments within the solvent-accessible regions while leaving the amyloid spine intact^12^. Consistent with this finding, SDS/PAGE analysis of six fibril samples previously incubated with PK revealed differences in the protomer PK-resistant core (Fig. 3a). Unexpectedly, the relative intensity of low molecular weight bands (<10 kDa) was higher for fibrils showing greater barostability/ThT binding capacities. To determine whether the results from PK-resistance could be explained by factors that affect the above-mentioned fibril pressure response, we related the kinetic parameter *k*_obs_^fast^ from the pressure-induced dissociation profiles to the densitometric analysis of bands. We report a progressive and non-linear loss of the lower PK-resistant bands (<10 KDa) with decreasing fibril barostability, that was concomitant with an increase of the proteolytically resistant core (gain of bands >10 KDa) (Fig. 3b). These results led us to conclude that fibril barostability decreases with the size of the amyloid spine. Accordingly, the sheet-to-sheet arrangement of extended amino acid stretches must be significantly more loosely packed, displaying focal cavities, compared to that involving short sequences.

### Amyloid fibrils generated by mutational variants M109I and E219K display profoundly different superstructure and seeding activity

**Figure 4:**
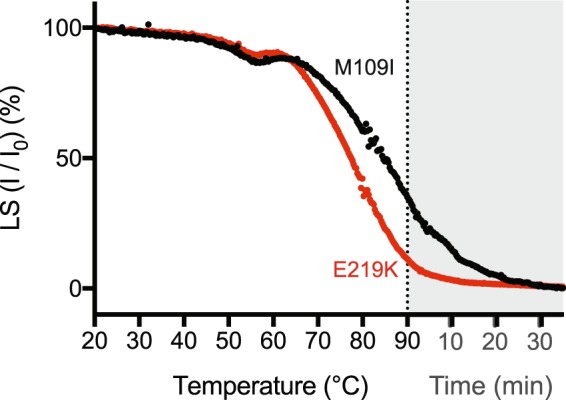
PrP fibrils show different temperature-induced dissociation kinetics. The extent of structural changes for M109I (black line) and E219K (red line) was recorded as a decrease in light scattering intensity after a gradual temperature increase. Temperature ramping experiments (1 °C/min) were performed starting at 20 °C up to 90 °C. The signal stability at 90 °C was monitored for another 30 min (grey rectangle). Data were normalized between 0 and 100 to easily compare the effects of temperature on the fraction of aggregated PrP.

In the next analyses aimed at further understanding the nature of these structural differences, we confined our analysis to the most divergent samples, M109I and E219K fibrils. To this end, static light scattering measurements were performed during temperature ramping (Fig. 4). The decrease in scattering intensity observed when the temperature increased from 20 to 90 °C indicated a reduction in the weighted average molecular weight of the sample. In agreement with what was observed during pressure treatment, the different persistence of scattering conformers at high temperatures when comparing both fibril samples (i.e. difference in the pseudo-melting temperature of 7.4 °C) show that the E219K fibril type was less stable than M109I. As for pressure studies, the temperature-induced kinetic profiles were found to be highly reproducible, even upon assaying two independent PrP fibrils obtained from a different stock of PrP WT sample.

**Figure 5:**
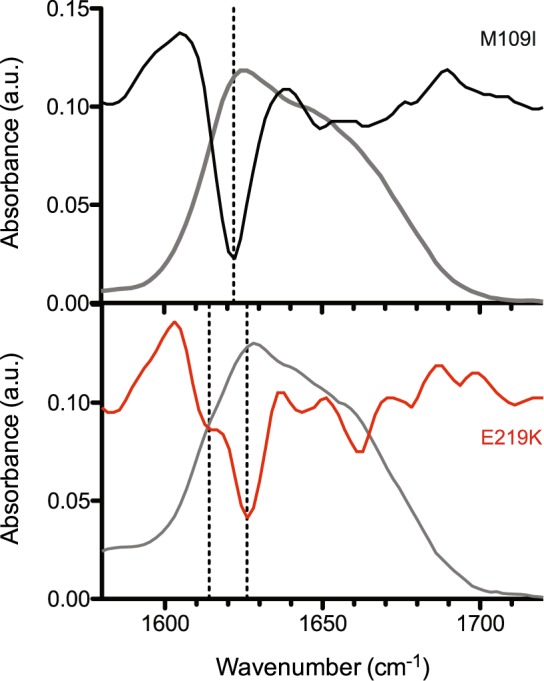
PrP fibrils show different secondary structure. ATR-corrected IR spectra (grey lines) and the corresponding second derivatives of M109I (black line) and E219K (red line) fibrils. Dashed lines depict positions of the IR component bands that correspond to the cross β-structure.

Next, attenuated total reflection–Fourier transform infra-red (ATR-FTIR) spectroscopy was used to monitor if the observed conformational features that distinguish M109I and E219K fibril polymorphism were accompanied by changes in the protomer secondary structure. Clearly different intermolecular ß-structures were observed (Fig. 5). Second derivative analysis of M109I fibrils showed a band at 1621 cm^−1^, assigned to β-sheets^13^. A positional blue shift of this band of about 5 cm^−1^ was observed for E219K fibrils. This suggest that M109I fibrils are endowed with β-sheeted structures involving either a larger number of strands or a more planar conformation, compared to E219K fibrils^14^. No high-wavenumber component (1685–1690 cm^−1^), generally found in anti-parallel β-sheets, was apparent in the spectra of both samples. In addition, the E219K fibrils contain intermolecular β-sheets (shoulder at 1614 cm^−1^), which could be assigned to a less stacked conformation^15^. The appearance of the component band at 1663 cm^−1^, generally assigned to β-turns, further reinforce the finding that E219K fibrils display greater dynamic flexibility compared to the M109I type.

**Figure 6:**
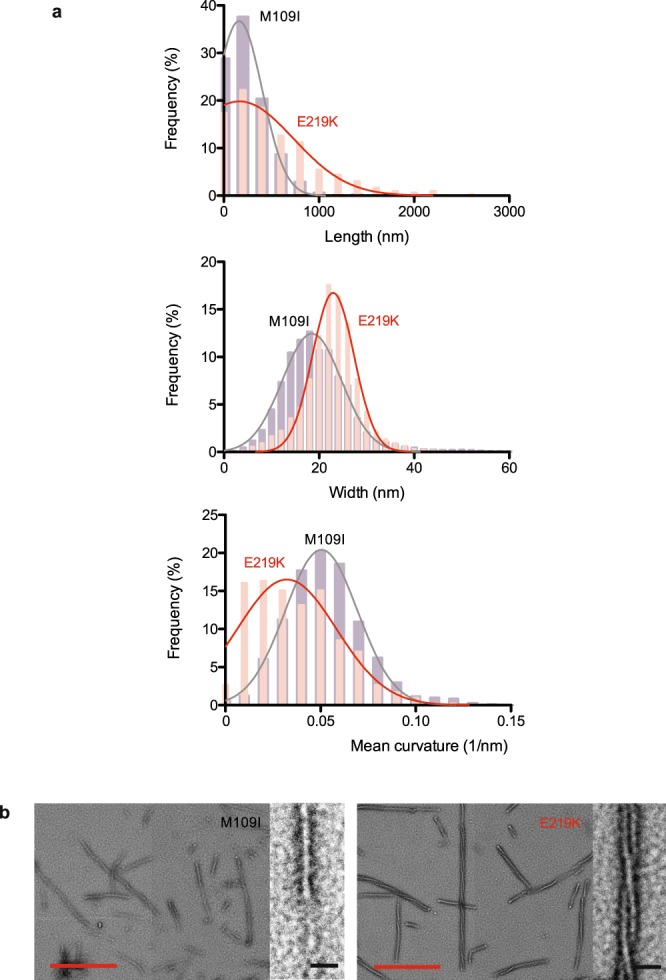
PrP fibrils show different morphology. (**a**) Frequency histograms of observed M109I (black) and E219K (red) fibril length, width and mean curvature data. (**b**) Negative-stained transmission electron micrographs of M109I and E219K PrP fibril forms. Two zoom-in images of the fibrils are shown. Note: slight defocusing was used to enhance contrast “relief” of the fibrils. Red scale bars, 0.5 μm. Black scale bars, 50 nm.

The macrostructural differences between M109I and E219K fibrils were further investigated by TEM (Fig. 6). We used an automatic quantification of three main parameters (fibril width, length and mean curvature) by image processing techniques. By providing a detailed characterization of each measurement distribution (Fig. 6a) we demonstrate that E219K fibrils show a length distribution that is larger, and present a wider and less curved shape compared to M109I fibrils. In addition, we found a higher order organization for E219K fibrils (Fig. 6b), which consist mainly of two or more protofilaments and display a clear helical twist that originates from the winding of the individual protofilaments around the axis of the fibril.

**Figure 7:**
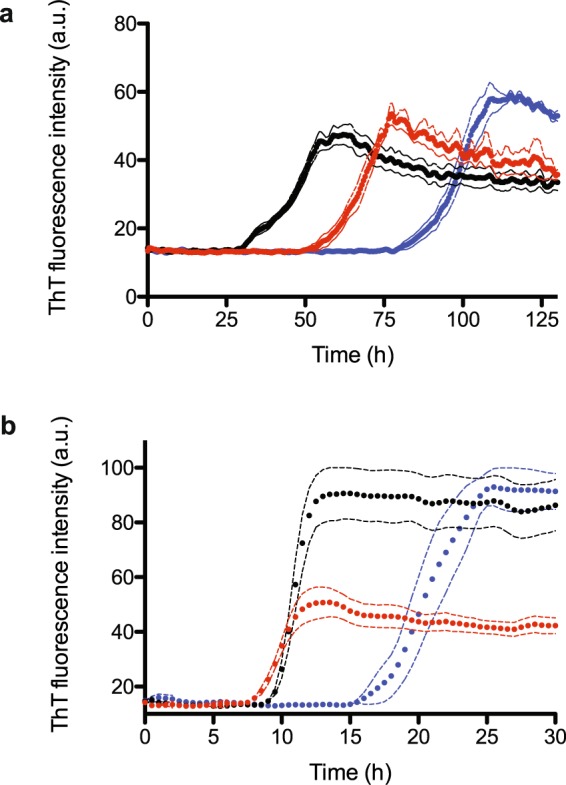
PrP fibrils show different seeding abilities. Fibrillisation kinetics of WT human PrP (**a**) and bank vole PrP (**b**). Reactions were unseeded (blue) or seeded with sonicated M109I (black) and E219K (red) fibril templates. Seeds were added at a 0.5:100 PrP fibrils: native PrP mass ratio. Amyloid fibrils growth was probed using microplate reader coupled to the standard ThT-based fluorescence assay. Averaged profiles (n = 6) of ThT fluorescence intensity.

Given that data presented so far strongly suggest that M109I and E219K fibrils structurally differ from each other, and suggest that they represent two independently end products of the PrP misfolding reaction, we wondered whether both fibril types differ in their *in vitro* seeding activity. Averaged profiles of ThT fluorescence intensity using full-length human PrP (WT) unseeded or seeded with the two freshly sonicated M109I and E219K fibril templates (added at a 0.5:100 PrP fibrils: native PrP mass ratio) are shown in Fig. 7a. Under the conditions of these measurements, and in the absence of any seeds, the process of *de novo* PrP fibrillation is rather slow with approximately 75 h-long lag-phase. In the presence of fibrils, this lag phase is decreased. We thus demonstrate that both M109I and E219K fibrils seed amyloid formation, albeit with different efficiencies (lag-phase of 25 and 50 h, respectively). These differences were further substantiated in homologous seeding assays (Supplementary Fig. 7). Nevertheless, and due to the inherent different onset of fibril formation of each PrP variant, their growth phase in seeded conditions started coincidentally (Supplementary Fig. 7). The higher seeding activity of the M109I fibrils compared to those of E219K was abrogated using bank vole PrP as substrate (Fig. 7b). The bank vole PrP (unseeded) shows an enhanced susceptibility to form fibrils with a fast-sigmoidal profile characterized by a 15 h-long lag phase. The lag phase was found to be similar and significantly shortened (approximatively of 10 h) in both seeded reactions. However, we obtained a considerably lower final ThT fluorescence plateau for the E219K seeded reaction, compared to that obtained using the M109I template. This is likely to originate from differences in ThT-binding capacities between both mother fibrils, and suggest a transgenerational imprinting of the fibril-specified ThT property using bank vole PrP as substrate, consistent with the fact that bank vole PrP appears to be a universal acceptor for prions that maintains strain specified properties^16^.

## Discussion

Not only the nature, but also the magnitude of the conformational rearrangements within the proposed spectrum of PrP quaternary structures, i.e. the breadth of prion strain diversity, is not well understood. Little is known about the influence of hydration and residue packing, which directly affect the volumetric properties of the protein^17^, in fibril polymorphism. Yet, this knowledge is crucial because it might govern the differences in prion disease pathology and clinical manifestation^18^.

To provide indirect information on this uncommon structural dimension (volume changes upon conformational transitions), in this work we investigated the effects of high pressure^19–21^ on seven fibril samples formed from wild-type and various single-point variants of full-length human PrP. The obtained experimental results allow us to delineate the wide superstructural landscape of human PrP fibrils in terms of their sensitivity to pressure.

A plethora of structurally distinct fibrils are predicted by the high-dimensional PrP folding energy landscape^22^. Recent available experimental data indicates that, while amyloid fibril spine is characterized by a common vast array of interbackbone hydrogen bonds, thermodynamically and conformationally unique fibrils can be selected and enriched by single point amino acid substitutions^23,24^. Here we provide various recombinant human PrP fibrils and report that the wide superstructural range of their assembly is evidenced by the marked differences in ThT-binding properties, proteolytic digestion, macroscopic appearance, and resistance to pressure dissociation. Particularly, we show that amino acid replacements associated to several natural *PRNP* gene polymorphisms fine-tune the conformational features of the resulting PrP fibrils. The most contrasting effects in fibril features were observed between PrP variants involving i) charged or polar side chains such as E219K and E211Q (hypothetical unfavourable burial upon assembly) and ii) hydrophobic residues such as M109I and G127V (favourable desolvation). Although the atomic structures of full length PrP amyloid fibrils are not yet available, several biochemical and biophysical studies suggest that the C-terminal domain of PrP forms the fibril spine, from which the non-structured N-terminus and the H1 must somehow project into the periphery^12,25–28^. On the basis of these studies and the present results, we also show that the location of the amino acid substitution in the monomeric PrP may also influence the conformational features of the fibril state. Fibrils formed from PrP variants bearing amino acid replacements in the C-terminal domain and specifically in H3 (E219K or E211Q) particularly diverge with those carried out in the N-terminal hydrophobic domain (M109I or G127V). The unstructured domain is known to be a critical modulator of the function of PrP^C^ in physiological and pathological conditions, as well as the target of many cofactors and antiprion compounds^29^. The obtained experimental results confirm the relevance of this region in PrP structural changes as well as its impact on the globular C-terminal rearrangements, and allow us to discuss further how single amino acid substitutions may account for multiple stable and conformationally distinct amyloid fibrils. Knowing which of the two parameters, the nature of the amino acid modification (i.e. ionisable/hydrophobic side chains) or the location of the replacement, plays a more important role in PrP fibril diversity remains to be determined.

Previously, we showed that upon pressure perturbation PrP oligomers and fibrils dissociate into monomeric PrP molecules that refold into the native conformation after pressure release^30,31^. These findings brought crucial knowledge on PrP structural changes that accompany the assembly process, not attainable with the classical approaches used to perturb the protein structure. Fundamentally, as a reaction is favoured under pressure when the volume change of the system is negative^32^, the observed pressure-induced disassembly must be dictated by a decrease in volume.

On the basis of a previous study on the molecular determinants of this volume loss upon protein unfolding^33^, the present observations on the markedly different fibril barostability are explained by the collapse of a different degree of localized defects, show up as cavities and not just by a disruption of the weak bond network and hydration of newly exposed residues. We demonstrate that the implicit volume change of the fibrils upon dissociation, evidenced by their pressure sensitivity, is inversely correlated with its ThT-binding capacities. The most barostable/compact M109I fibril type shows the highest ThT fluorescence, whereas the most barosensitive/voluminous E219K shows the minimum fluorescent yield. This suggests that cavities that provide the appropriate binding environment for ThT to induce the characteristic ThT fluorescence^34^ do not correspond, at least exclusively, to the void volume (trapped inside the quaternary structure) that accounts for the fibril behaviour under pressure. In addition, fibril barostability shows a roughly inverse correlation with the size of PK-resistant core. This suggests that packing defects increase with the size of the interface regions (spine) formed by the assembly process, here characterized as protease-resistant folds. The small size of the peptides obtained from the proteolytic digestion of the most barostable/compact M109I fibril is congruent with previous studies showing a lower molecular weight PK-resistant PrP fragment in the brains of in spontaneously ill transgenic mice overexpressing bank vole PrP with the M109I polymorphism^35^, compared to the brains of FFI and CJD(E200K) patients. Thus, the various pressure response observed for the fibril variants arise from the void spaces in the self-associating protomer interfaces, rather than packing defects in the protomer interior. This is in agreement with the finding that a protein assembly interface has about twice the number of cavities relative to the tertiary structure^36^. However, because of the small sample size (7 fibril samples), the general relevance of our results has to be considered with caution. Further investigations on the amyloid properties (barostability *vs* ThT binding or PK-resistance) of an additional range of PrP fbrils would be important for thoroughly understanding these relationships.

At the molecular level, the fact that PrP fibrils can be dissociated by high temperature and by pressure indicates that the density of interactions (temperature) and packing (pressure) within the amyloid fold is lower than that observed in the native protein. This is supported by our previous results obtained by applying isopycnic density gradient centrifugation (equilibrium separation), which show that PrP^Sc^ is found in fractions of lower density than PrP^C^. This observation is consistent with the presence of packing defects that might arise during the assembly of the PrP protomers to form the PrP^Sc^ assemblies. Furthermore, a previous study using DSC suggests that the dissociation of amyloid fibrils involves a much lower net balance of interactions (lower enthalpy change) and a higher area of hydrated surface (lower apparent heat capacity changes) for the protomer in the fibrillar state relative to the native protein^37^.

## Conclusion

Therefore, one may speculate that fibril barostability, besides offering a fundamental and highly informative probe of its volumetric physics, might also be associated with its seeding-dependent propagation. However, further studies are necessary to attribute this new structural dimension observed with recombinant PrP fibrils to specific prion strains and make predictions about their *in vivo* activities. Given that we can now link specific barostabilities to single PrP fibrils morphotypes, and that prion strains generate different types of aggregates that can be differentiated by their intrinsic density^38,39^, the experiments described here should enable new approaches to define how the degree of compaction in prions dictate the mechanism by which the amyloid spread between cells and in tissues.

## Methods

### Recombinant PrP

Full-length human PrP and six variant forms corresponding to PRNP gene polymorphisms were generated using the QuickChange site-directed mutagenesis kit (Stratagene), produced in *Escherichia coli* and purified as described previously^40^. M109I represents the natural polymorphism in bank vole populations, and G127V, M129V, N171S, E211Q, E219K correspond to naturally occurring variants of the human prion protein. Full-length bank vole PrP were also obtained using the same protocol. Purified monomeric PrPs were stored lyophilized.

### Formation of Amyloid Fibrils (manual assay)

PrP amyloid fibrils were formed using the manual setup protocol described previously by^11^. Briefly, the lyophilized protein was dissolved directly in 50 mM MES buffer, pH 6.0 at 1 mg/ml. This solution was kept on ice before starting the experiment. To prepare a 500 μl reaction, the following reagents were mixed in a conical plastic tube: water (90 μl), GdnHCI (6 M, 200 μl), MES buffer (0.5 M, pH 6.0, 10 μl) and finally the PrP stock solution (300 μl). The solution was mixed by gently pipetting to avoid introducing air bubbles. Typically, a 10-ml reaction was made in a 15-ml conical centrifuge tube. The tube (arranged horizontally on the plate surface) was incubated with continuous orbital shaking at 30 r.p.m. (16 mm amplitude) at 37 °C. Fibril formation was monitored using a ThT binding assay^11^. For this assay, aliquots were withdrawn and diluted into 10 mM Na-acetate buffer, pH 5.0 to a final concentration of PrP of 0.3 μM. Then ThT was added to a final concentration of 10 μM. Samples were dialyzed in 10 mM sodium acetate, pH 5.0. Then fibrils were collected by ultracentrifugation for 45 min at 228147 × g using a Beckman Optima TL100 Ultracentrifuge and TLA-100.3 rotor, and resuspended in 10 mM sodium acetate, pH 5.0. A washing step was performed by repeating the ultracentrifugation and resuspension steps. All concentrations given for fibrillar PrP refer to the respective equivalent monomer concentration. For a second passage of homologous seeding, sonicated mother fibrils (1 min in an ultrasonic water bath (Advantage Lab AL04-04)) were added to freshly prepared PrP solutions at 0.5:100 PrP fibrils: native PrP mass ratio.

### Fibrillisation Kinetics and Seeding Capacity (semi-automated assay)

Fibrillisation kinetics of unseeded or seeded with different amyloid templates (seeds added at a 0.5:100 PrP fibrils: native PrP mass ratio) were probed using a microplate reader (SAFAS Xenius XML) coupled to the standard ThT-based fluorescence assay (ThT final concentration of 10 μM). 96-well black microplates were used with three Teflon beads (3/32 in. diameter Marteau et Lemarié, Pantin, France) per well. The reaction volume was 160 μl. Kinetic experiments were carried out at 37 °C under agitation. We performed intermittent shaking cycles, consisting of 1 min of shaking at 600 r.p.m. followed by a 1-min incubation break. In order to assess reproducibility of aggregation kinetics, 6 microplate wells were filled with each sample for simultaneous measurements. To potentially normalize fibril size, amyloid templates in 1.5 ml conical plastic tubes were subjected to sonication for 1 min in an ultrasonic water bath (Advantage Lab AL04-04) and assumed to have approximately the same number of growth sites per weight of fibril.

### Transmission Electron Microscopy (TEM)

Samples were deposited on Formvar carbon-coated grids, negatively stained with freshly filtered 2% uranyl acetate, dried and viewed using a JEOL 1200EX2 electron microscope (JEOL USA, Inc, Peabody, USA).

### Automatic quantitative morphological analysis of amyloid fibrils

A new method of image analysis was developed for the automatic treatment of TEM images. Fibrils were segmented by a Maximum Entropy thresholding method and then the Fast Marching skeletonization is applied to detect the fibril centreline. We then use a tracking method based on a Bayesian estimation of the local value and gradient of the pixel intensity^41^, which allows us to estimate the local width, length and curvature. The intricate overlapping and branching structures are identified based on the angles between fibril segments.

### Fluorescence Measurements

Fluorescence measurements were performed at 20 °C using a FP-6200 fluorimeter (Jasco France, Bouguenais, France) or a microplate reader (SAFAS Xenius XML). Aliquots of each protein sample at a final concentration of PrP of 0.3 μM were incubated with 10 μM thioflavin T (ThT) in 10 mM Na-acetate buffer, pH 5.0 for 1 min at room temperature before fluorescence measurements. ThT emission spectra were recorded after excitation at 450 nm. The excitation and emission slit widths were 4 nm.

### Proteinase K Digestion

Fibrils were treated with proteinase K (proteinase K to PrP ratio of 1:100) at 37 °C for 1 h. 5 μg of PrP fibrils were thus digested using 0.05 μg of PK, and the digestion reaction was stopped with 2 mM phenylmethylsulfonyl fluoride. Samples were heated in denaturing sample buffer (60 mM Tris– HCl, 2% SDS, 5% β -mercaptoethanol, 2.25 M urea) at 95 °C for 10 min and separated on 12% Criterion™ XT Bis-Tris Precast Gels (Bio-Rad) followed by silver staining. Quantification of protein-band intensities was performed by densitometric analysis using Image Lab software (Biorad).

### High Pressure Treatment

PrP fibrils were diluted in the same buffer to a final protein concentration of 10 μM and placed in 5-mm diameter quartz cuvettes closed at the top with flexible polyethylene film that was kept in place by a rubber O-ring. Pressure jumps consisted of rapid (30-s) changes of the atmospheric pressure to obtain a final pressure of 360 MPa. A pressurization cycle at 360 MPa was performed by maintaining the pressure for approximatively 8 h at 25 °C and by decompression of the sample to atmospheric pressure.

### Light Scattering Measurements under High Pressure

Light scattering measurements were carried out using an Aminco Bowman Series 2 fluorescence-spectrophotometer (SLM Aminco) modified to accommodate a thermostated high pressure optical cell. Protein disaggregation was followed by monitoring the changes in scattered light at 300 nm (4-nm slit) and excited at 300 nm (4-nm slit).

### Estimation of the Kinetic Parameters

The relaxation profiles of the fibril structural reactions were fitted to double exponential decays, according to Equation (1),

1 $$I(t)={I}_{0}+A(1-{e}^{-{k}_{obs(1)}t})+B(1-{e}^{-{k}_{obs(2)}t})$$

where *I*(*t*) and *I*_0_ are the fluorescence intensities at time *t* and at time 0, *A* and *B* are the phase amplitudes, and *k*_*obs*_ is the measured apparent rate constant at the final pressure *P*.

### Static Light Scattering Temperature Ramping

Static light scattering temperature ramping experiments were performed on a homemade device using a 407-nm laser beam in 2-mm quartz cuvettes and in a thermostated cell holder starting at 20 °C and up to 90 °C. Light-scattered signals were recorded at a 112° angle. Signals were processed with a homemade MatLab program. For both types of experiments, PrP fibril concentration was fixed at 1 μM (monomer equivalent). The cuvette was removed from the holder at high temperature and gently inverted to mix the sample. No change in the scattered light was observed suggesting the complete dissociation of the protein assembly.

### FTIR-ATR Spectroscopy

Spectra were recorded on a Bruker Equinox 55 spectrometer equipped with a MCT detector, using a spectral resolution of 4 cm^−1^. The spectrometer was continuously purged with dry air. The ATR cell with a silicium crystal was filled with the deuterated 10 mM MES buffer at pD 5.5 and a reference spectrum was collected. The cell was then filled with the deuterated M109I and E219K fibril samples at the respective monomer concentration of 44 µM and 38 µM. Interferograms were recorded as for the reference spectrum and transformed by ATR correction to the absorption spectrum. Deuterium oxide (D_2_O) was obtained from Eurisotop (France). Second derivative analyses of Amide I’ band in the spectral range 1600–1700 cm^−1^ gave the fixed wave numbers of each component band assigned to secondary structure element for each PrP fibril type.

## Supplementary information

Supplementary Info (legends and figures)

## Data Availability

The datasets generated during and/or analysed during the current study are available from the corresponding author on reasonable request.
