# Supplementary material for: Pressure Reveals Unique Conformational Features in Prion Protein Fibril Diversity
Source: Sci Rep. 2019 Feb 26;9:2802. doi: 10.1038/s41598-019-39261-8 (PMC6391531; doi:10.1038/s41598-019-39261-8)

Supplementary Information

Pressure Reveals Unique Conformational Features in Prion Protein Fibril Diversity

Joan Torrent[1,2*], Davy Martin[1], Sylvie Noinville[3], Yi Yin[4], Marie Doumic[4], Mohammed Moudjou[1], Vincent Béringue[1] and Human Rezaei[1]

[1] Institut National de la Recherche Agronomique, UR892, Virologie Immunologie Moléculaires, F-78350 Jouy-en-Josas, France

[2] present address: Université Montpellier, Montpellier, F-34095 France ; Inserm, U1198, MMDN, Montpellier, F-34095 France ; EPHE, Paris, F-75007, France.

[3] Sorbonne Universités, UPMC Univ Paris 06, CNRS, UMR8233, MONARIS, Université Pierre et Marie Curie, F-75005 Paris, France

[4] Sorbonne Universités, Inria, UPMC Univ Paris 06, CNRS, UMR7598, Lab. J.L. Lions, F-75005 Paris, France

*To whom correspondence should be addressed: Joan Torrent, Université Montpellier, Montpellier, F-34095 France ; Inserm, U1198, MMDN, Montpellier, F-34095 France ; EPHE, Paris, F-75007, France. Tel.: (33) 4 67 14 33 87; Fax: (33) 4 67 14 92 95; E-mail: joan.torrent@inserm.fr

SUPPLEMENTAL FIGURE LEGENDS

Supplementary Figure 1.

Soluble native PrP variants have similar secondary structure.

Circular dichroism spectra in the aromatic region of the various soluble native PrP forms.

Supplementary Figure 2.

“Mother” and “daughter” generations of M109I and E219K PrP fibrils maintain the same ThT-binding capacities upon one round of homologous seeding.

Seeds were added at a 0.5:100 PrP fibrils: native PrP mass ratio. ThT fluorescence emission maxima of each set of data is the mean value ± S.E of quadruplicate measurements

Supplementary Figure 3.

Pressure-induced dissociation of PrP fibrils.

Pressure-induced PrP fibril dissociation after a rapid increase of pressure to 300 MPa. The structural change kinetics were recorded as a decrease in light scattering. Fibril fraction was calculated from the amount of resolubilized PrP rescued after the pressure treatment, as judged from the absorbance at 280 nm of the supernatants obtained after removing fibrils by centrifugation. Dashed lines, linear fits to the data. The kinetic profiles of M109I and E219K PrP fibrils are indicated in black and red, respectively. All other profiles are denoted in blue. Note: only one out of every 9 points are shown to facilitate visualization of the kinetic traces.

Supplementary Figure 4.

ThT-binding capacities of the PrP fibrils correlate with the extent of dissociation induced by pressure.

Changes in the fibril fraction was calculated from the amount of resolubilized PrP rescued after the pressure treatment, as judged from the absorbance at 280 nm of the supernatants obtained after removing fibrils by centrifugation of the PrP fibrils as a function of PrP fibril barostability. The dotted line shows the best fit to a linear equation (r=0.71).

Supplementary Figure 5.

High reproducibility of the pressure and temperature-induced kinetic profiles upon assaying two independent PrP fibril preparations obtained from a different stock of PrP WT sample.

(A) Pressure-induced PrP fibril dissociation after a rapid increase of pressure to 300 MPa. The structural change kinetics for PrP WT were recorded as a decrease in light scattering. Fibril fraction was calculated from the amount of resolubilized PrP rescued after the pressure treatment, as judged from the absorbance at 280 nm of the supernatants obtained after removing fibrils by centrifugation. a.u., arbitrary units. (B) The temperature-induced dissociation kinetics of PrP WT was recorded as a decrease in light scattering intensity after a gradual temperature increase. Data were normalized between 0 and 100 to easily compare the effects of temperature on the fraction of aggregated PrP.

Supplementary Figure 6.

Full-length gel shown in Figure 3A.

Red line shows the cropped location**.**

Supplementary Figure 7.

PrP fibrils show different seeding abilities.

Fibrillisation kinetics of M109I (A) and E219K (B) human PrP. Reactions were unseeded (black) or seeded (red) with the homologous fibril templates (homologous seeding). Seeds were added at a 0.5:100 PrP fibrils:native PrP mass ratio. Amyloid fibril growth was probed using microplate reader coupled to the standard ThT-based fluorescence.

Sup. Figure 1

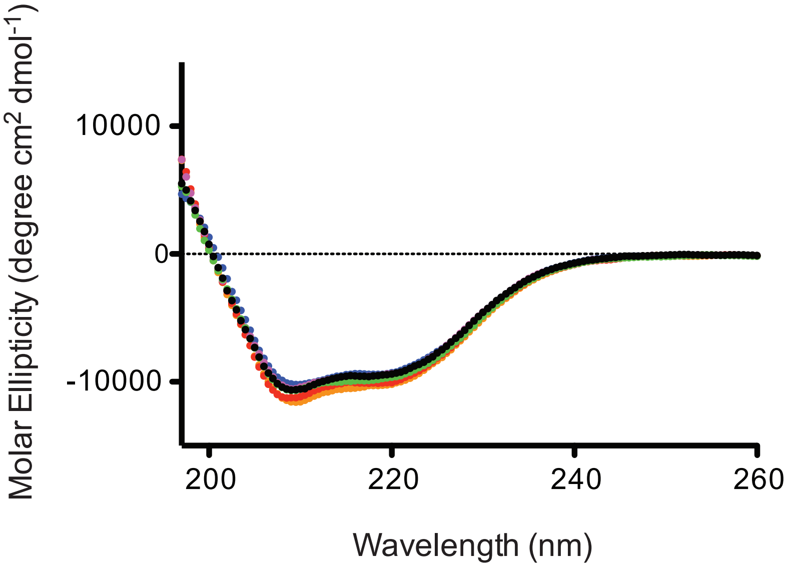

Molar Ellipticity (degree cm2 dmol-1)
10000
0
-10000
200
220
240
260
Wavelength (nm)

Sup. Figure 2

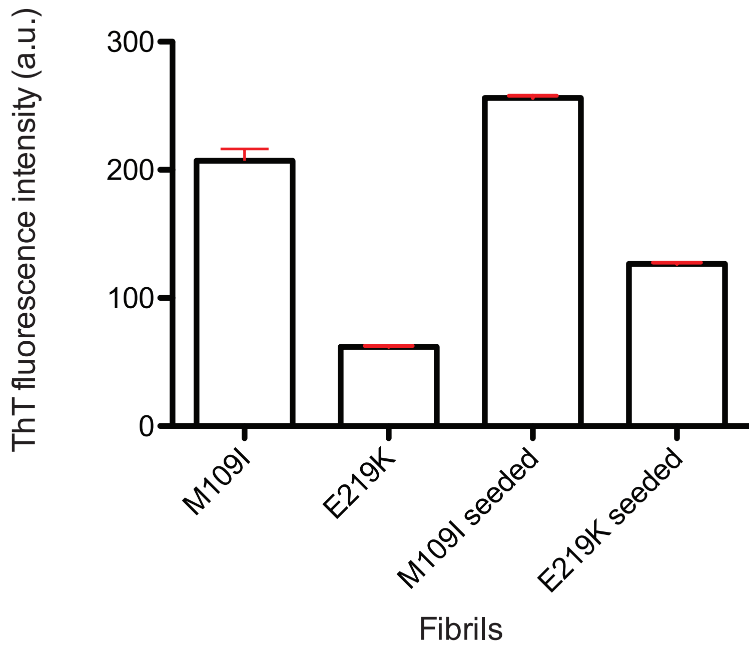
ThT fluorescence intensity (a.u.)
300
200
100
0
M109I
E219K
M109I seeded
E219K seeded
Fibrils

Sup. Figure 3

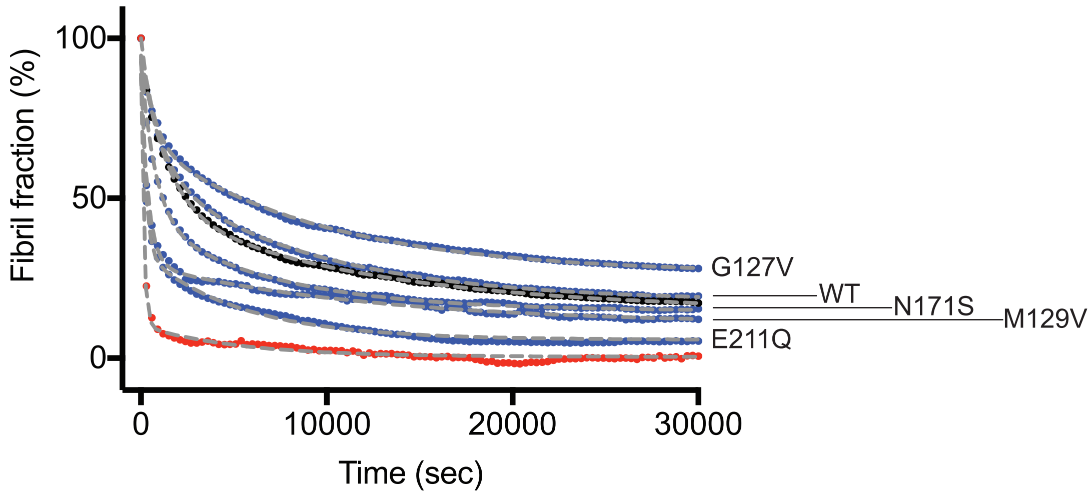

Fibril fraction (%)
100
50
0
0
10000
20000
30000
Time (sec)
G127V
WT
N171S
M129V
E211Q

Sup. Figure 4

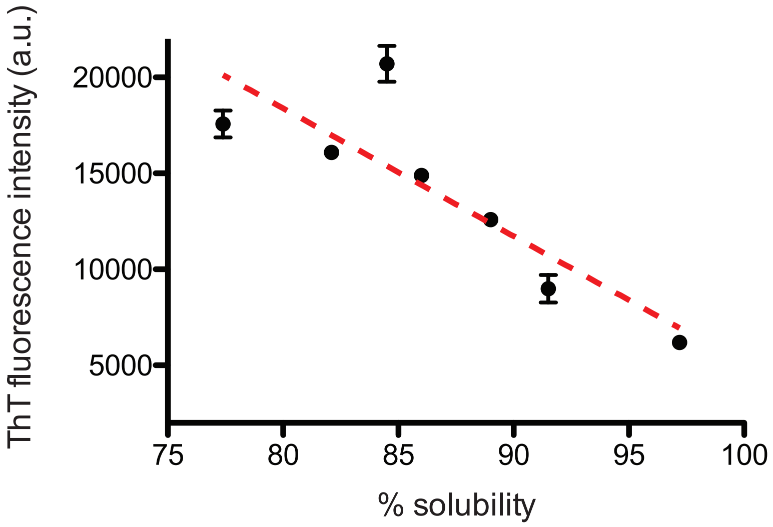

ThT fluorescence intensity (a.u.)
20000
15000
10000
5000
75
80
85
90
95
100
% solubility

Sup. Figure 5

a

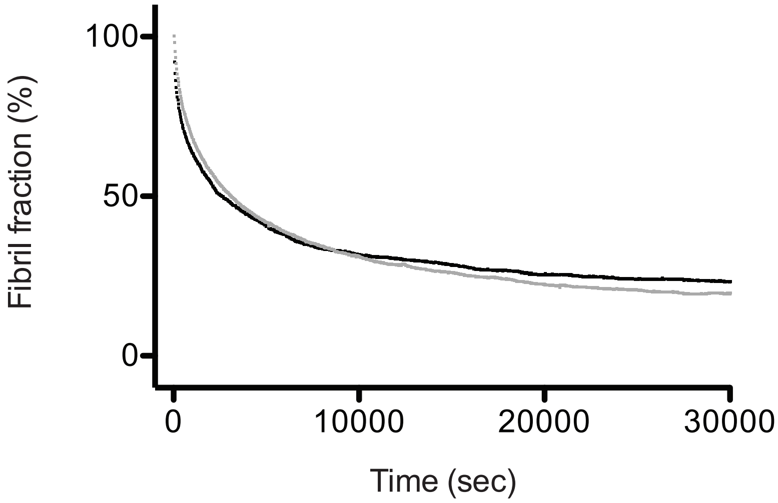

b

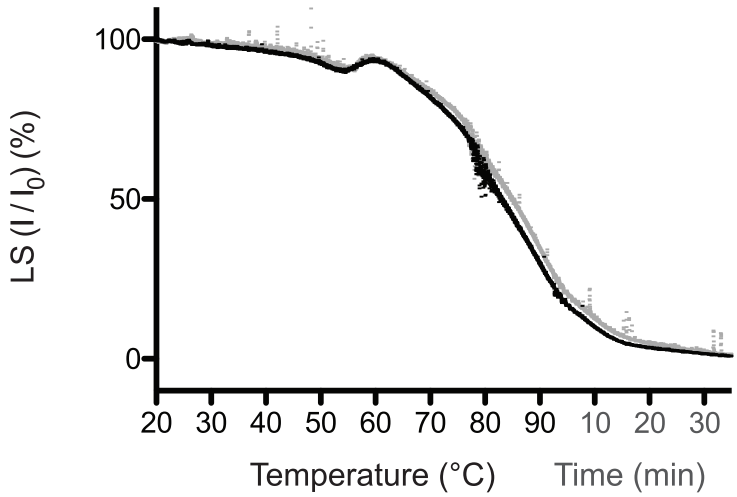

Sup. Figure 6

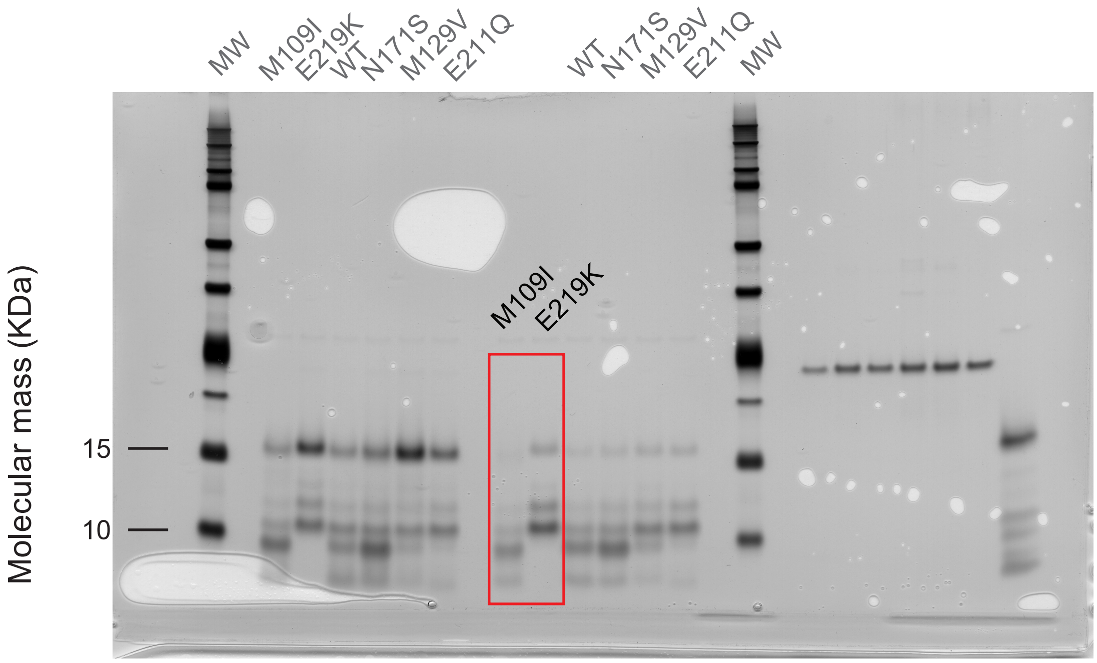

MW
M109I
E219K
WT
N171S
M129V
E211Q
WT
N171S
M129V
E211Q
MW
M109I
E219K
Molecular mass (KDa)
15
10

Sup. Figure 7

a

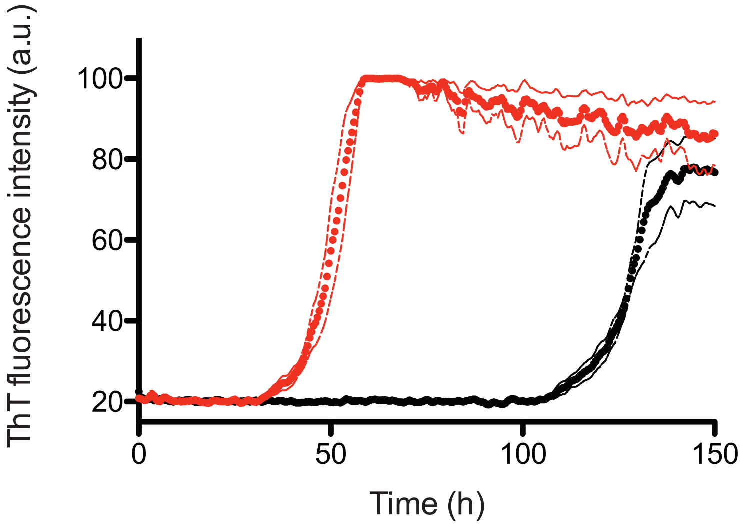

b

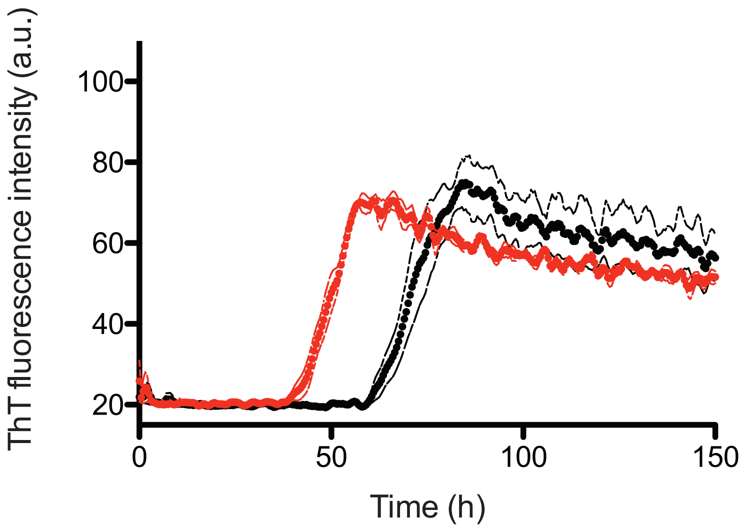

Supplement: Supplementary file 1 — Supplementary Info (legends and figures) [file 41598_2019_39261_MOESM1_ESM.pdf]
